# Spatio-temporal variation of noise pollution in South Paris during and outside the COVID-19 lockdowns

**DOI:** 10.1038/s41598-024-51305-2

**Published:** 2024-01-17

**Authors:** M. A. Abdmouleh, S. Dahech

**Affiliations:** UFR GHES UMR 8586 du CNRS (PRODIG), Faculté Sociétés Et Humanités, Université Paris Cité, Paris, France

**Keywords:** Environmental sciences, Environmental impact

## Abstract

Noise pollution is one of the major environmental problems of contemporary societies. In urban areas, road transport is the main source impacting the spatio-temporal variation of air quality. This work aims to analyse the spatio-temporal variability of the noise level in the 13th arrondissement during peak hours, focusing on the comparison of noise levels between the COVID-19 lockdown and non-lockdown days. This paper provides data that could be used to evaluate noise mitigation options. Mobile surveys, using EXTECH 4017764 sensors, were used to cover 272 points. In this work, the mobile measurements use the median of instant noise levels measured every 5 s over a 5-min period during the peak period. This study confirms that road traffic appears to be the determining factor in noise pollution. The noise levels calculated in 2020 show a strong spatio-temporal variability explained by the proximity of the emission sources, but also that the noise level decreases by 6–10 dB (A) during the lockdowns. Indeed, near the main roads, 57–63 dB (A) are recorded during the lockdowns, compared to 67–72 dB (A) outside the lockdowns. Mainly the number of vehicles contribute to a large part of the noise level, to which the noise of construction sites can occasionally be added as in the south-eastern part of the study area.

## Introduction

The city concentrates multiple sources of noise, hence the scarcity of pleasant noise environments in urban areas. Noise is strongly linked to the urbanisation process and the development of industry and transport^1,2^. In order to ensure a moderate level of noise, it is important to find the elements that influence this phenomenon. Urban noise is an integral part of the environment in large cities and is considered a source of nuisance. Indeed, it is the first source of complaints and one of the first sources of conflicts, at work, between neighbours, and between local authorities and users^3^.

Noise can severely affect the health of individuals and disturb the tranquillity of populations^4,5^ causing high blood pressure and ischaemic heart disease^2,6,7^, or reduced efficiency at work or school^8^. Noise can also cause hearing, sleep, and learning disorders^9–12^. Pregnant women may be more sensitive to noise pollution because of their susceptibility to environmental stressors^13,14^. Similarly, noise sensitivity can have a significant influence on psychiatric disorders such as anxiety and depression^15^. "Noise pollution is at the heart of the problems of modern society"; it changes with time, place and perceptio^16^. Furthermore, research has shown that not only the intensity of noise pollution but also the duration of exposure to traffic noise has a negative impact on health^17–19^.

Recent studies have analysed the spatial variation of noise levels, identified the affected population, and presented solutions to mitigate noise^13,20–22^.

Noise nuisance subsided as the COVID-19 pandemic spread through Europe a few months after it first appeared in China. Indeed, several governments and public authorities around the world responded by introducing severe restrictions on the movement of people and goods to contain the phenomenon. The management of the COVID-19 epidemiological emergency throughout the country led to a radical transformation of the noise environment, especially in large cities^23^.

A study conducted by Bruiparif, based on 18 stations in the Ile-de-France region, showed an average decrease of 5.9 dB LDEN (A) (Level day-evening-night) during the first lockdown^24^. In Paris, the first lockdown was more severe than the second. At noon on 17 March 2020, the first national lockdown began, that extended to last nearly 2 months. France initiated easing lockdown measures on 11 May. But all schools reopened for 2 weeks in late June. The restrictions of the second lockdown, from 30 Octobre to 15 december 2020, were less strict, with schools and many businesses enduring open^24^. The third lockdown took place from April 3 to June 30. After a month, decontamination was gradual, with the opening of schools. On June 30, the curfew will be lifted once and for all, and depending on the local health situation.

Other results on noise during the lockdowns can be cited: in Barcelona, the noise pollution level decreased by 9 dB LDEN (A) after 1 week of lockdown^25^, reductions of up to 8 dB LDEN (A) were measured on road networks in Athens, and a reduction of 5.4 dB LDEN (A) was observed at 11 sampled sites in London^26–28^. In Paris, following the implementation of lockdown measures, road traffic noise levels decreased by an average of 7.6 dB LDEN (A)^24^.

One of the purposes of the paper is the comparison of noise level recorded during and outside of lockdowns. Also, this paper presents the spatial distribution of noise generated by road transport and construction sites during and outside the lockdowns in the 13th arrondissement of Paris, during peak hours (morning and evening) by mobile survey method. In parallel, the paper study the spatial and temporal distribution of the number of vehicles in circulation during and outside the lockdowns.

Compared with works assessing the changing urban sound environment during the COVID-19 lockdown period, mainly in Europe, our study covers 272 locations compared with only 8 in Athens^28^, 5 in Madrid^27^ and 11 in London^26^. The high density of measurement points (approx. 30 points per km^2^) enables us to pinpoint the spatiotemporal variability of noise on a fine scale, and thus determine the noise level to which the population is exposed in all neighborhoods.

## Study area

**Figure 1:**
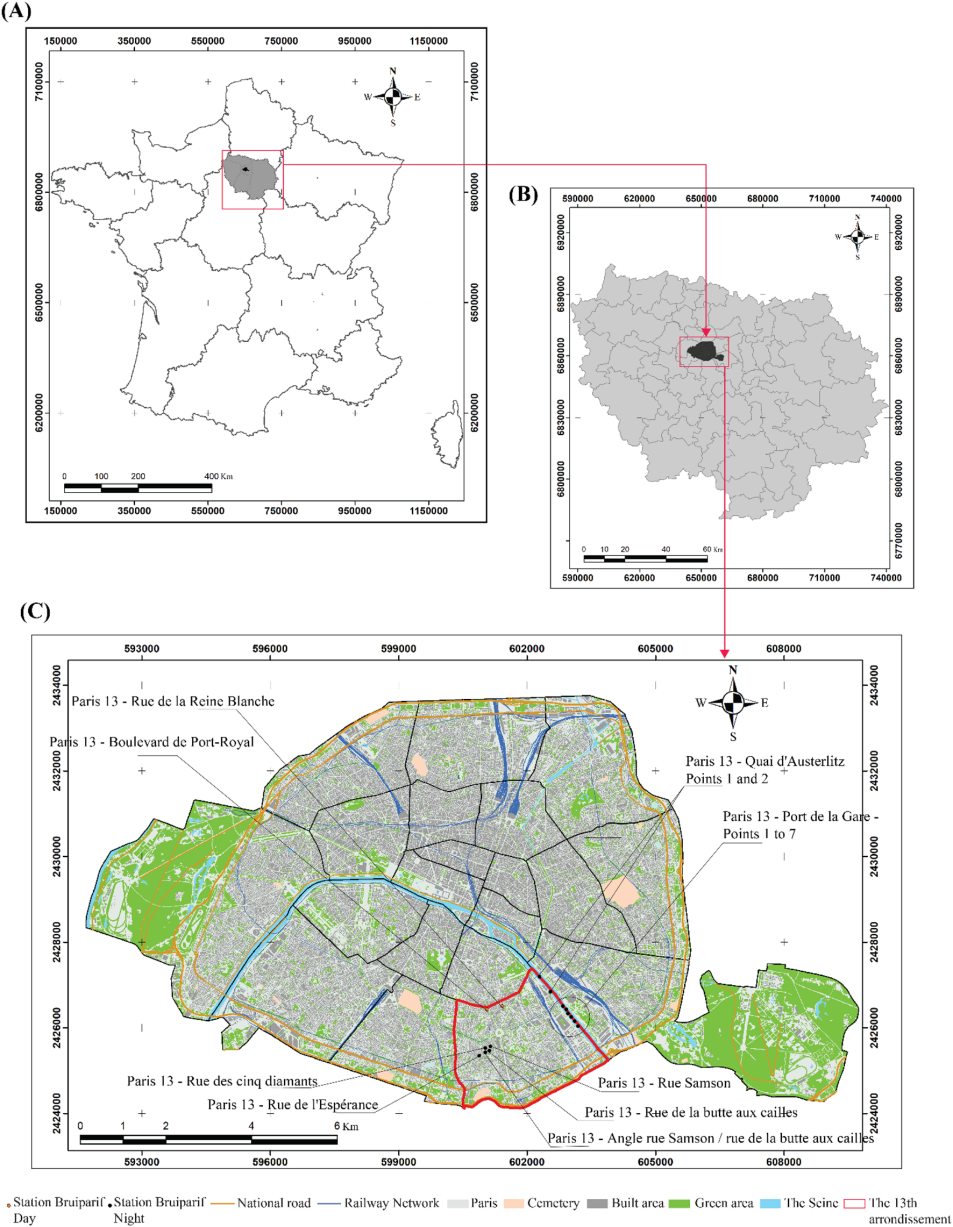
Location of the study area (**A**,**B**) and the Bruitparif noise pollution monitoring stations (**C**) (source: www.data.gouv.fr).

In the north of France, the Île-de-France region is a source of economic dynamism on a national, European, and global scale (Fig. 1). This region accounts for half of the jobs in the French economy^29^. According to the latest report by the French National Institute of Statistics and Economic Studies (INSEE)^24^, the Île-de-France region counts 12,213,447 inhabitants. The region is characterised by its economic dynamism and concentrates various industrial and tertiary activities with dense road traffic due to the multiple movements of the population and goods that converge there. It represents 6.1 million jobs^30^.

For this study, mobile surveys are performed in the 13th arrondissement as part of an ongoing PhD. According to the latest INSEE census (2016), the average population density in the 13th arrondissement is 25,392 inhabitants/km^2^ for an area of 7.2 km^2^. The older districts built in the 1970s are denser, such as Olympiade, Place Nationale, Place d'Italie and Place Pinel.

These districts are characterised by a road network with a tight and homogeneous north–south and east–west grid. It is composed of ring roads (boulevard de Port-Royal, Arago and Saint-Marcel, boulevard des Fermiers Généraux, rue de Tolbiac), main radial roads (avenue des Gobelins, avenue d'Italie, quai de la Seine, boulevard de l'hôpital) and secondary roads (rue Jeanne d'Arc, rue de Patay, avenue de Choisy, rue de Bobillot, rue de Glacière, rue de Nationale, rue de l'Admiral Mouchez, rue de la Santé, etc.). The Place d'Italie is the major nodal point of this arrondissement^31^.

Property values are secondarily dependent on noise and the environment in general. As an example, a decrease in property value due to neighbourhood noise is estimated at 10–20% of the value of a property exposed to neighbourhood noise (shop, café, restaurant, bar, etc.)^32^. Mobile sources seriously contribute to the increase in noise pollution in Paris. The Environmental Noise Prevention Plan (PPBE) brings together the actions carried out by the City of Paris and its partners (Bruitparif, Préfecture de Police, etc.) to reduce the exposure of Parisians to road noise^33^.

## Methods and data

The purpose of collecting data for the study was to compare noise levels outside of lockdowns (pre and post lockdowns), with noise levels during lockdowns.

The study considered mobile attended measurements were carried out during the 2nd and 3rd lockdowns and outside of the lockdowns.

**Figure 2:**
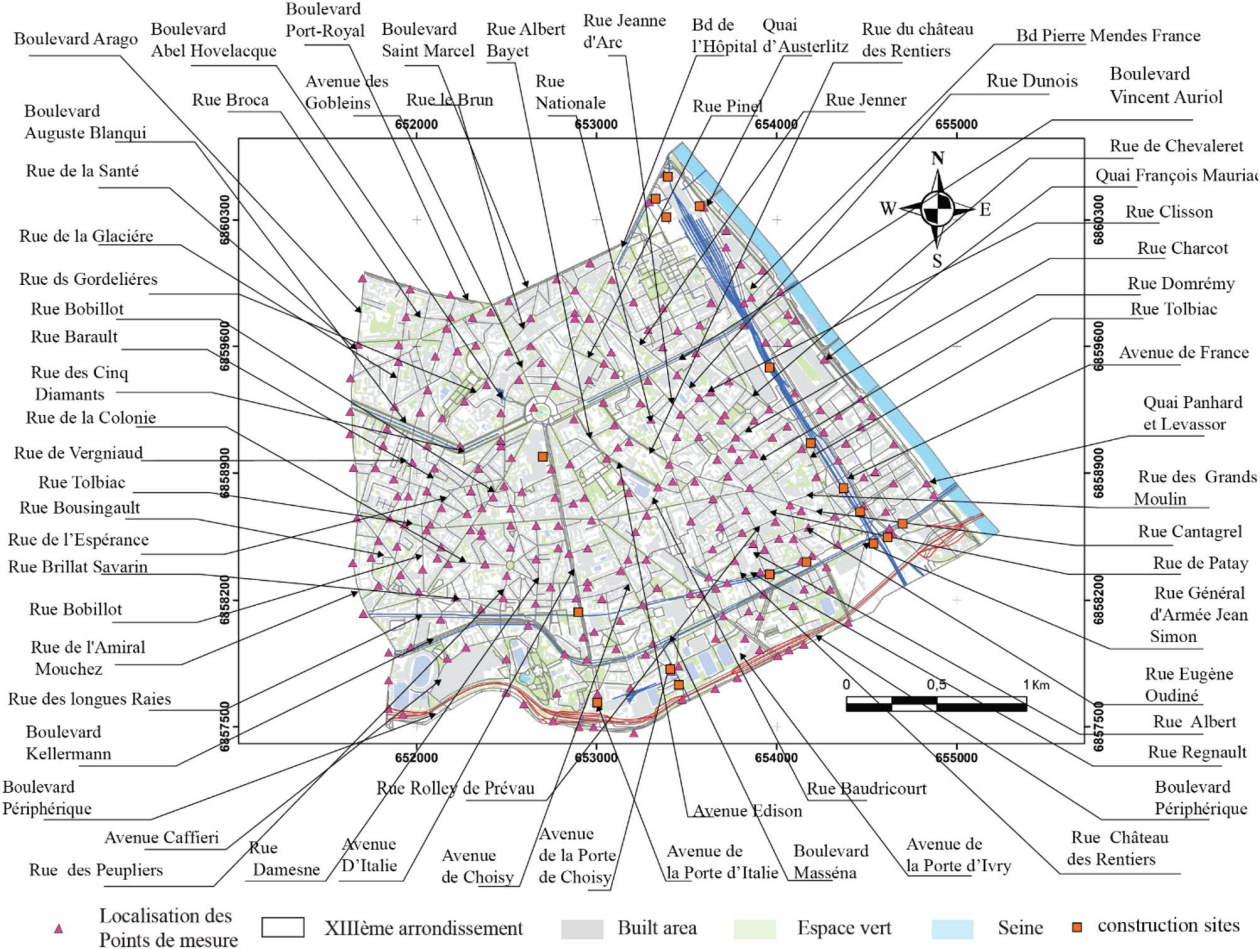
Location of measurement points and the roads where vehicle traffic flow was recorded and cross-referenced (source: www.data.gouv.fr).

The mobile attended measurements were carried out at locations approximating a grid with a spacing of 100 m across the 13th Arrondissement with a total of 272 measurement points. The grid included important roads, intersections and construction sites and points were selected to represent good coverage of the study area and differing urban environment characteristics. Figure 2 shows the location of the points.

A total of 70 measurement sessions were carried out with 34 sessions during lockdown and 36 outside of the lockdown. Each session measured at 20 points. Sessions were done during the lockdown between 3 November 2020 and 26 November 2020 (2nd lockdown) and between 3 March 2021 and 3 May 2021 (3rd lockdown). Sessions outside of the lockdown were carried out between July 2020 and February 2022. Measurements were carried out during both the morning peak (7.30 am–9.30 am) and the evening peak (5 pm–7.30 pm).

At each point the following data was collected: a 5 min noise measurement, the number of vehicles passing on the road, wind speed and the orientation and size of arterial roads relative to the prevailing wind.

The noise level was logged every 5 s during the 5-min measurement using an EXTECH 407764 sound level meter, giving a total of 60 noise levels logged at each point. The measurements were compared with Bruitparif station for calibration. In total there was 84,000 noise levels recorded across 70 sessions of measurements at 20 points in each session.

The median noise level from the levels recorded at each point was selected for the analysis. This was to minimise the influence from peak noise levels such as car horns. Noise levels were excluded where they were affected by extraneous noise or where the effects of weather such as wind were influencing the data. Noise from mechanical plant was not included in the measurements.

The number of vehicles was counted by taking note during the campaign of the 5-min stops. Since traffic is relatively homogeneous during peak hours, the values are then multiplied by 12 to obtain an estimate of road traffic/hour.

Data is presented in 2 forms—the 75th percentile dots and the interpolated map. They essentially convey the same information in different ways. The first method locates sites with high values, while the second gives an idea of the different sound levels measured at the 272 points and interpolated to cover the entire study area.

The analysis has used the noise levels equal to or above the 75th percentile of the measurements during and outside the 2nd and 3rd lockdowns. Whilst the 75th percentile represents noisier sections; it was chosen due to its ability to create readable noise maps as plotting all of the points would have made the map unreadable. The 75 percentile is also close to the limit value not to be exceeded during the day (70 dB (A)).

Noise maps were produced to present the spatial distribution of measured noise levels using the inverse distance weighted interpolation (IDW) tool in ArcGIS Geostatistical Analyst toolbar. IDW interpolation is a widely used technique for mapping environmental variables. It is an exact and convex interpolation method that fits only the continuous model of spatial variation^34–36^. Our approach is inspired by work based on interpolation from fixed station data^36,37^. these interpolation methods have not been tested at fine scales, and further studies are needed to validate the conclusions of this work.

The basic principle of IDW interpolation is to use a set of weighted linear combinations of sample points, it relies on both statistical and mathematical methods to create surfaces and predictions based on weighting as a function of distance from the base data^38^. The weighting factors decrease as the distance to the network node increases^37,39^.

In this study, 272 observation points with a spacing of 100 m are used to produce maps showing the noise distribution during and outside the lockdowns. The results are cross-referenced with the number of vehicles.

### Spatio-temporal variability of daily noise levels in the morning period: analysis of the 272 measurement location data

**Figure 3:**
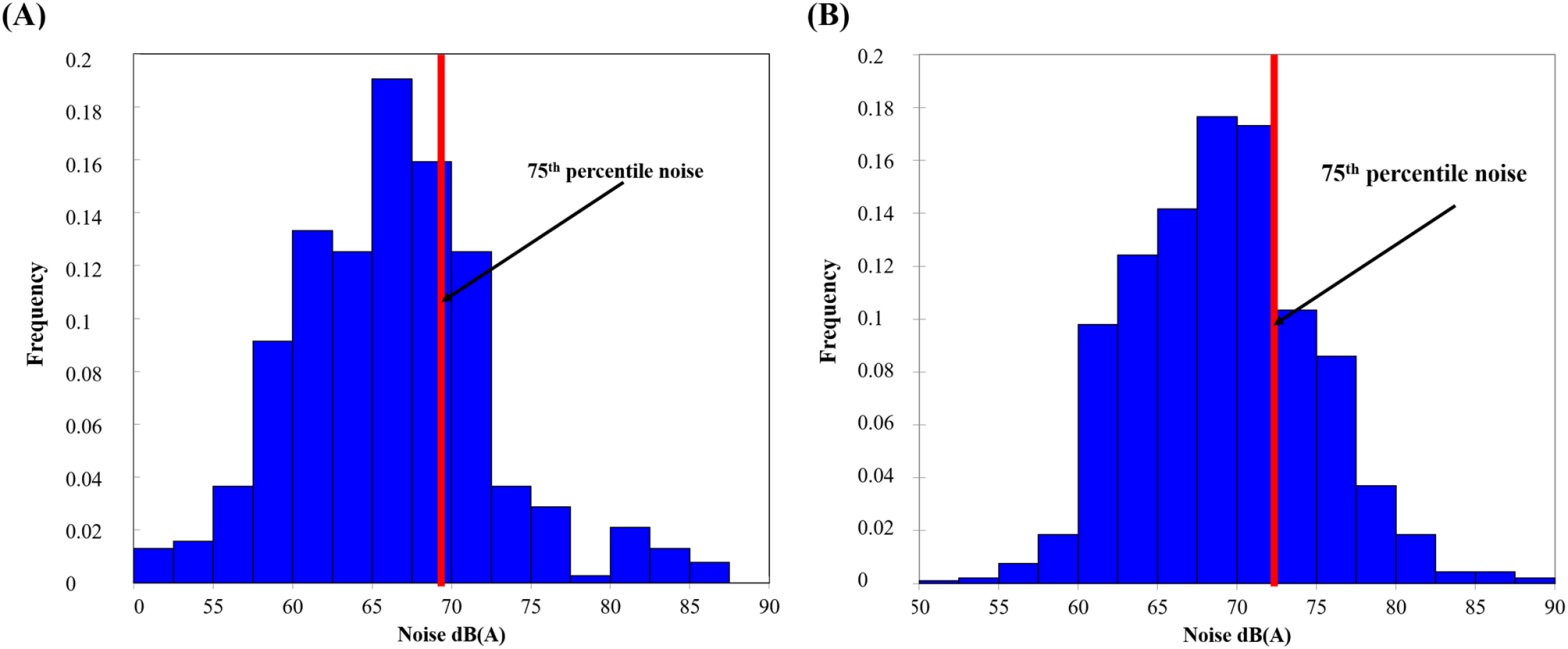
Frequency of noise levels in the 13th arrondissement exceeding the 75th percentile in the morning during (**A**) lockdown and (**B**) non-lockdown periods between 2020 and 2022 (time step 5 s, measurements by EXTECH sensors, number of survey campaigns: 34 during the lockdown, 36 outside the lockdown).

The 75th percentile during and outside the lockdown are of the order of 69 dB (A) and 72 dB (A), respectively (Fig. 3). The noise level is higher by 4–6 dB (A) at all points outside the lockdown, but the difference between the 75th percentile is 3 dB (A).

The values exceeding the 69 dB (A) percentile during the lockdown are evenly distributed in the southern part of the 13th arrondissement, recorded in traffic-dominated areas. The values exceeding 69 dB (A) were recorded mainly in two areas: the first is between the rue Quai Panhard et Levassor and the boulevard du Général d'Armée Jean Simon.

**Figure 4:**
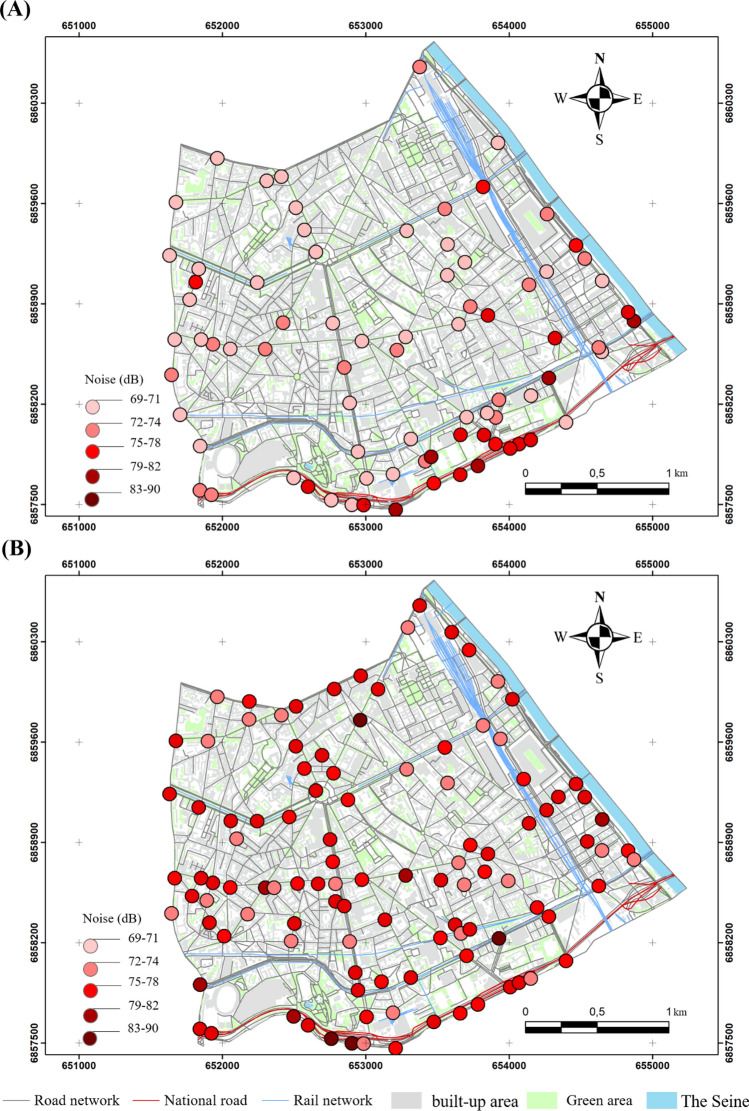
Noise distribution in the morning in the 13th arrondissement in 2020 and 2021, (**A**) during the lockdowns, (**B**) outside the lockdowns (average of medians); measurements carried out between September 2020 and February 2022, time step 5 s, EXTECH sensor, only values exceeding the 75th percentile are presented here).

The second includes the Boulevard Périphérique and Masséna and the avenues of Porte de Choisy and Porte d'Ivry with levels varying from 70 to 80 dB (A) (Fig. 4A). This configuration is completely different outside the lockdown, where the values become higher. They exceed 75 dB (A) in most of the crossings, except for a few observations ranging from 72 to 74 dB (A) at the boulevard Arago, rue Patay, rue Danois and rue Regnault (Fig. 4B). However, those ranging from 83 to 90 dB (A) are more concentrated on the Boulevard Périphérique, Boulevard de l'Hôpital and Boulevard Masséna (Fig. 4B).

This uneven distribution of noise levels in the 13th arrondissement of Paris is mainly explained by the variability of road traffic during and outside the lockdowns. The reduction in noise levels was greater in active areas than in traffic-dominated areas proportional to the decline in the number of vehicles.

**Figure 5:**
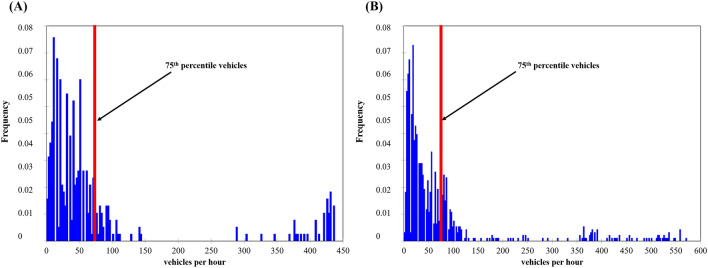
Number of cars counted at each of the 272 measurement locations described in the 13th arrondissement and the 75th percentile in the morning for the periods (**A**) during the lockdown and (**B**) outside the lockdown between September 2020 and February 2022 (personal traffic counting; number of campaigns 34 during the lockdown, 36 outside the lockdown).

The 75th percentile was calculated to locate the relatively high number of vehicles. It corresponds to 60 cars/5 min for the lockdown period while it amounts to 69 cars/5 min outside the lockdown (Fig. 5).

**Figure 6:**
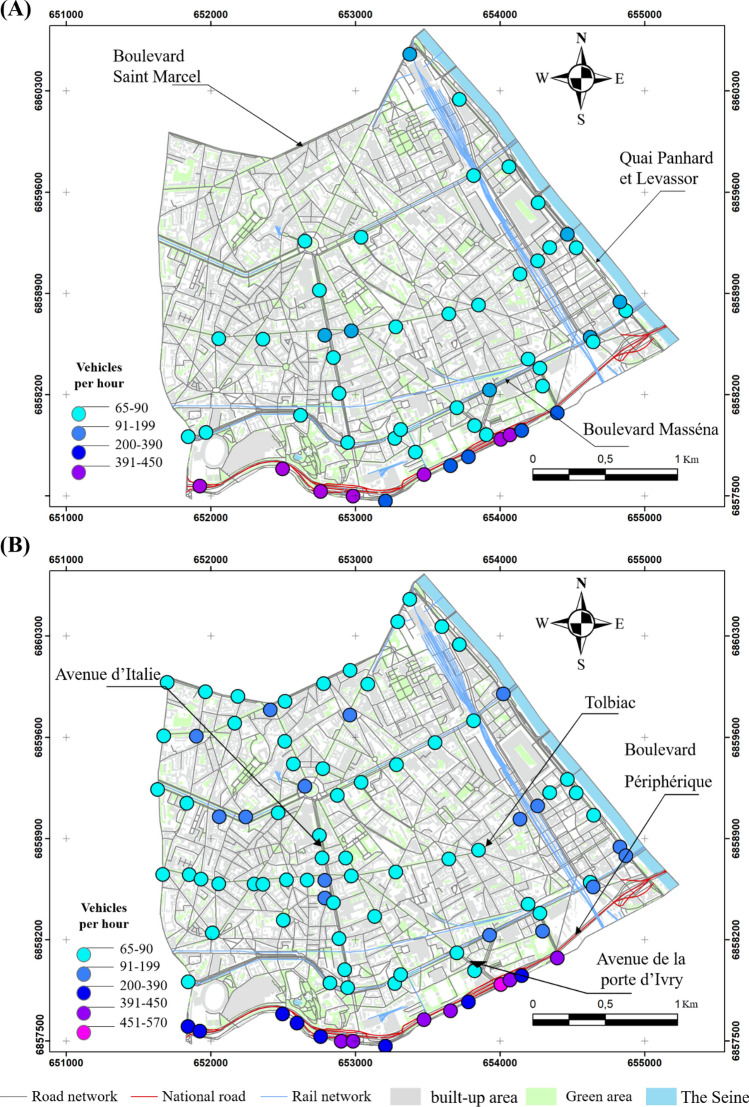
Distribution of the number of vehicles per 5 min in the morning in the 13th arrondissement in 2020 and 2022 during the lockdown (**A**), outside the lockdown (**B**) (average of 5 min per point, validated two or three times, campaigns conducted between September 2020 and February 2022; only the values exceeding the 75th percentile are presented here).

The results also indicate a notable spatial variability, even during the COVID-19 lockdown, where on some days 200 cars/5 min were recorded during peak road traffic hours, such as at Tolbiac, Avenue d'Italie, Boulevard Masséna, Quai Panhard et Levassor and avenue de France considered as active areas. But 320 cars/5 min are counted at the Boulevard Périphérique, considered as traffic-dominated areas (Fig. 6). Road traffic flow during the lockdown could be explained by the adoption of remote working by the government as a solution to limit the spread of the virus. According to a survey conducted by the Institut Paris Région, the proportion of remote workers among active workers doubled during the lockdown period, reaching 39%, compared to 18% before the lockdown^40^. This new work organisation has changed the way people work and has positively impacted the environment in general and air quality and noise in particular^40^.

Outside the lockdown, the situation changes and the number of cars increases, clearly exceeding 350 cars/5 min at Tolbiac, quai Panhard et Levassor, Avenue d'Italie, Boulevard Saint-Marcel. On the other hand, the record numbers reach more than 420 cars/5 min at the Boulevard Périphérique (Fig. 6B). Outside the lockdown, lower values were observed, sometimes on main roads such as Quai d'Austerlitz, Boulevard Saint-Marcel, and Boulevard Masséna.

### Spatio-temporal variability of daily noise level during the evening peak hours

**Figure 7:**
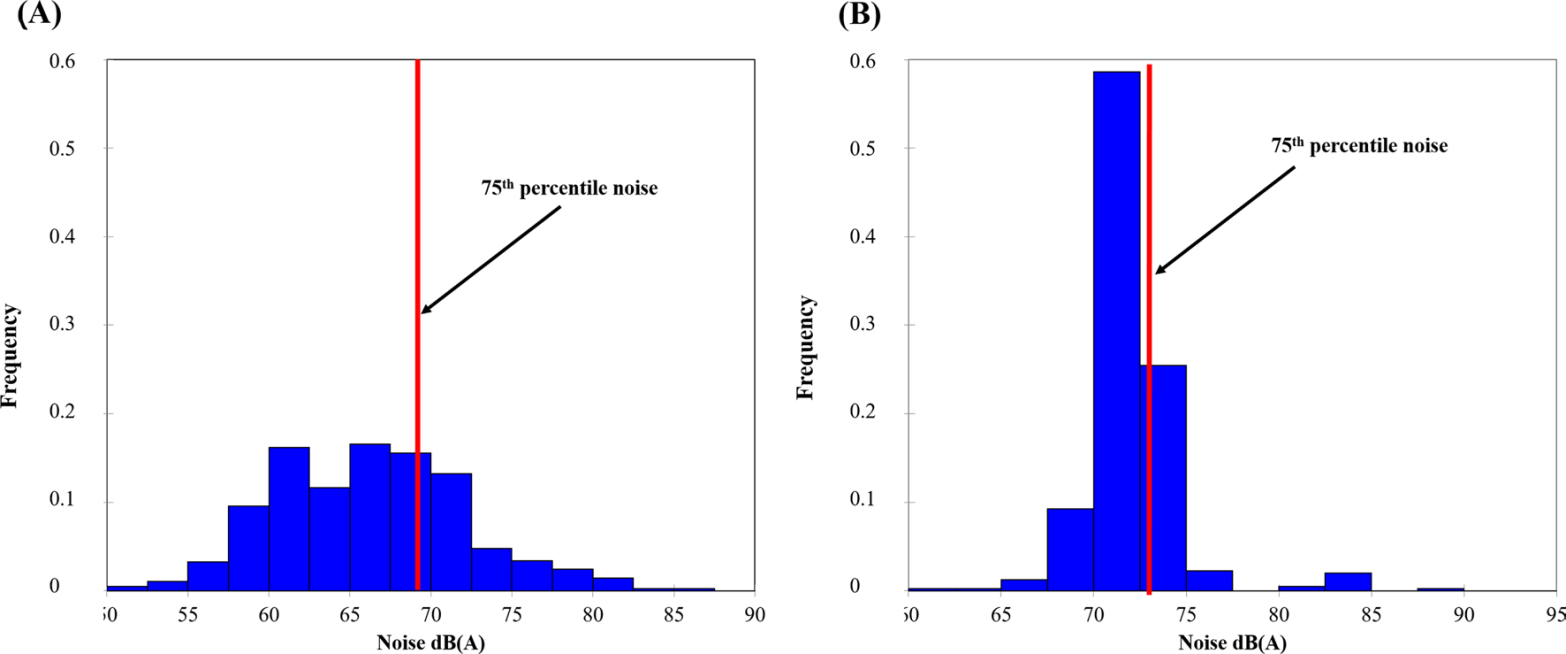
Frequency distribution of noise levels in the 13th arrondissement in the evening for the periods (**A**) during the lockdown and (**B**) outside the lockdown between 2020 and 2022 (time step 5 s, measurements by EXTECH sensors, number of campaigns 34 during the lockdown, 36 outside the lockdown).

The 75th percentile corresponds to 68 dB (A) for the lockdown period while it rises to 73 dB (A) outside the lockdown (Fig. 7).

**Figure 8:**
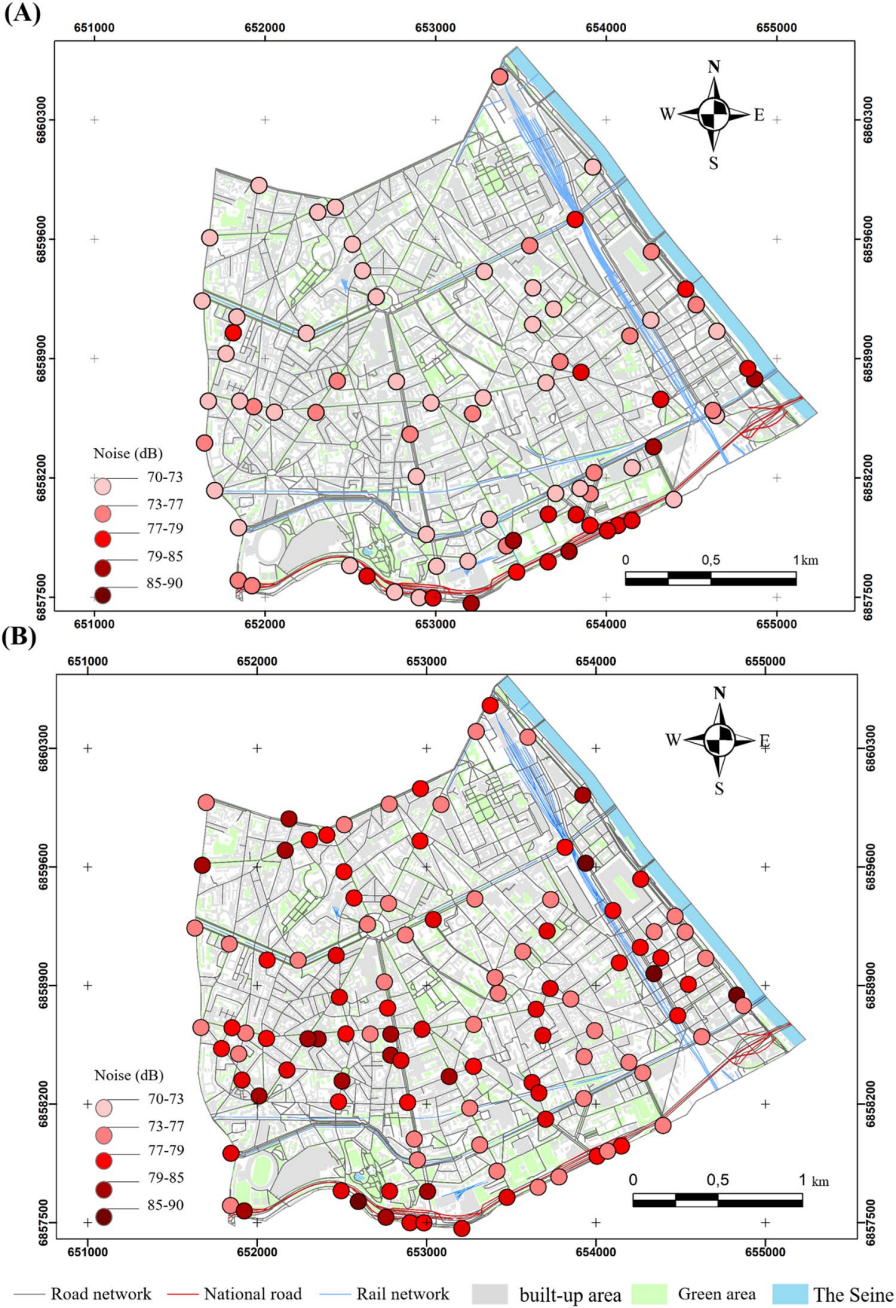
Spatial distribution of noise during the evening in the 13th arrondissement in 2020 and 2022 during the lockdown (**A**) and outside the lockdown (**B**) (average of 5 min per point, validated three times, measurements carried out between September 2020 and February 2022, 5-s recording step, EXTECH sensor, only values exceeding the 75th percentile are presented here).

**Figure 9:**
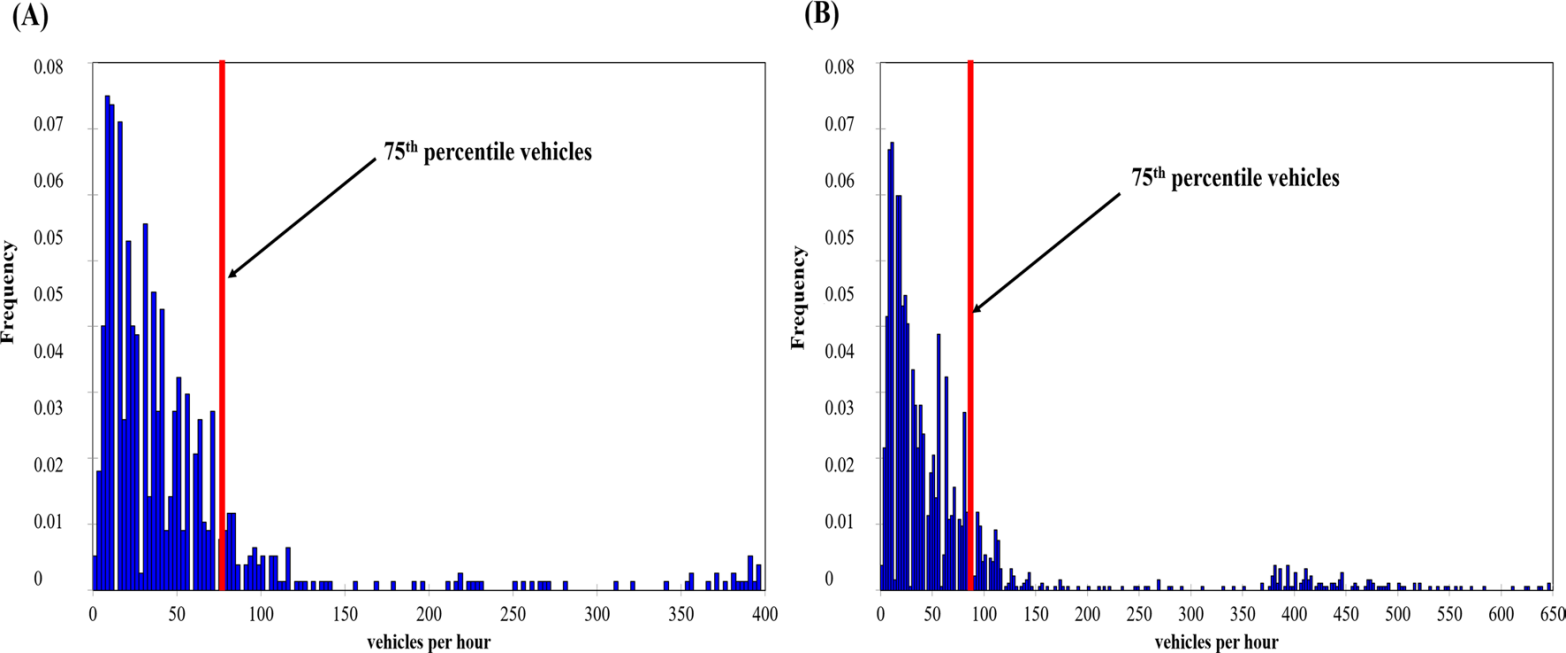
Frequency of the number of cars in the 13th arrondissement exceeding the 75th percentile in the morning during the (**A**) lockdown and (**B**) non-lockdown periods between 2020 and 2022 (5-s time step, EXTECH sensor measurements, number of campaigns 40 during the lockdown, 36 outside the lockdown).

**Figure 10:**
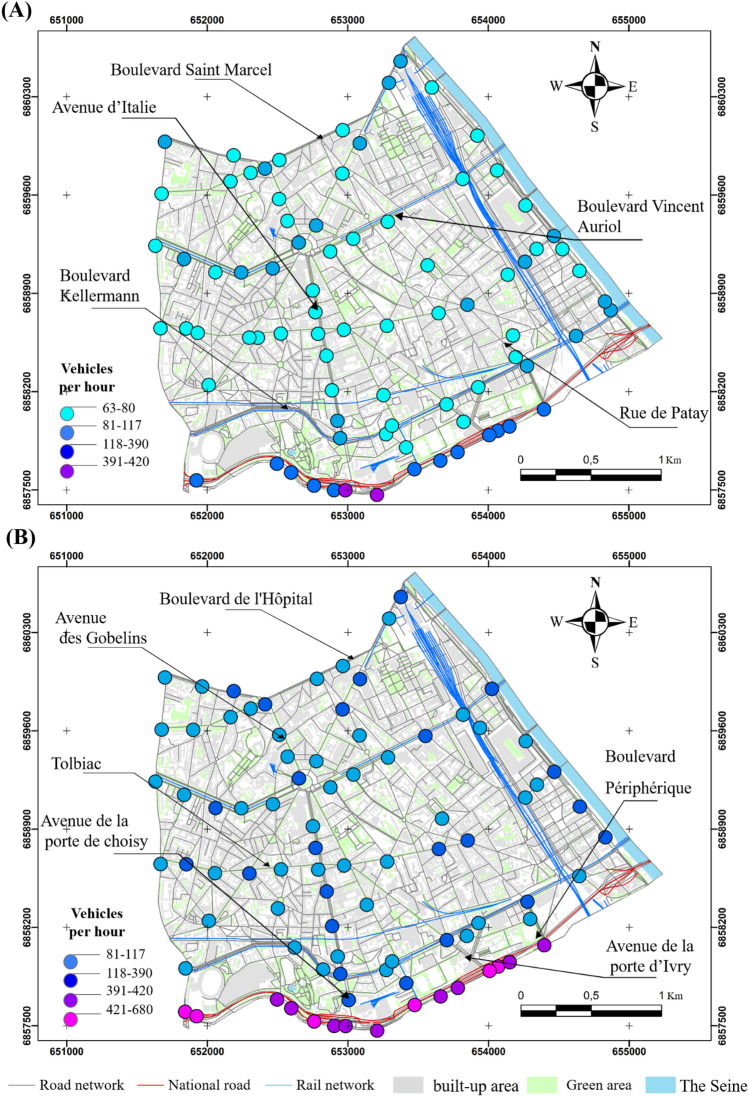
Distribution of the number of vehicles per 5 min during the evening in the 13th arrondissement in 2020 and 2021 during the lockdown (**A**), outside the lockdown (**B**) (average of 5 min per point, validated three times, campaigns conducted between September 2020 and February 2022; only the values exceeding the 75th percentile are presented here).

The results of the semi-itinerant survey campaigns confirm the correlation between road traffic density and noise (Figs. 7, 8, 9, 10). The distribution of noise pollution during lockdown shows two clusters, one to the east at the quai Panhard et Levassor and the other to the south along the Boulevard Périphérique and Avenue de la Porte d'Ivry, and at the Boulevard Masséna. These clusters record values up to 80 dB (A) (Fig. 8A). This is approximately the same pattern observed during the morning traffic peak.

Outside the lockdown, levels exceeding 73 dB (A) are evenly distributed in the southern part of the 13th arrondissement, resulting in a cluster in the east varying from 73 to 79 dB (A) with some exceedances, particularly near the Avenue de France, Boulevard Auguste Blanqui, and Boulevard Masséna.

A cluster in the middle of the arrondissement ranging from 80 to 85 dB (A) is located between the Avenue d'Italie, Avenue des Gobelins, Place d'Italie and Boulevard Auguste Blanqui. Another cluster in the southern end of the arrondissement with sound levels ranging from 86 to 90 dB (A) is located near the Boulevard Masséna, Avenue d'Italie, Boulevard Périphérique (Fig. 8B). Values exceeding 90 dB (A) were recorded between the intersection of the Avenue d'Italie and Tolbiac (Fig. 8B).

The variation of the noise is explained by the number of vehicles. The situation is almost similar to the morning phase. The 75th percentile corresponds to 63 cars/5 mn for the lockdown period, whereas it is 69 cars/5 mn outside the lockdown period, i.e. a slight increase compared to the morning phase. Indeed, "Parisians returning to the capital experience more difficulties in the evening (11% of the drive in traffic jams) than in the morning"^40–42^.

The results also show a majority of values ranging from 55 to 80 cars/5 min on the main roads such as rue de Tolbiac, Avenue d'Italie, Boulevard Vincent Auriol, and Boulevard Saint-Marcel. The number of vehicles increases at some intersections like Place d'Italie, or between Avenue de France and Tolbiac. The highest numbers are still found on the Boulevard Périphérique with an average of about 300 cars/5 min (Fig. 9A), both in the morning and in the evening, resulting in a noise level of about 85 dB (A). On secondary roads such as Boulevard Kellermann, Rue Nationale, and Rue Patay, the noise level drops considerably as the number of cars does not exceed 70 cars/5 min (Fig. 10A).

Outside the lockdown, the number of vehicles increases along the main roads where it exceeds 100 cars/5 min, as in Tolbiac, Avenue des Gobelins, Avenue d'Italie, and Boulevard de l'Hôpital (Fig. 10B). The traffic becomes more congested compared to the lockdown near the secondary roads by rising from 120, during the lockdown, to more than 250 cars/5 min as in Boulevard de l'Hôpital, Rue Patay, Avenue de la Porte de Choisy. The situation deteriorates further on the main roads where more than 500 cars/5 min are recorded, mainly on the Boulevard Périphérique, and at the major junctions such as between the Avenue de la Porte d'Ivry and the Boulevard Périphérique (Fig. 10B), where the noise level can occasionally exceed 90 dB (A) and slightly exceeds the peaks observed in the morning.

In addition to road traffic, other sources influence potentiallly the measurements of noise in the 13th Arr. including, community noise e.g. bars/cafes, and commercial noise e.g. a/c units. These noise sources are more prevalent in the late afternoon at points off the Boulevard Périphérique.

For example, spatial distribution of noise during the evening, shows an increase in noise near bars/cafes along the Avenue de France after work.

### Interpolation of the point data and impacts of the vehicle numbers for the morning and evening during and outside the lockdown

**Figure 11:**
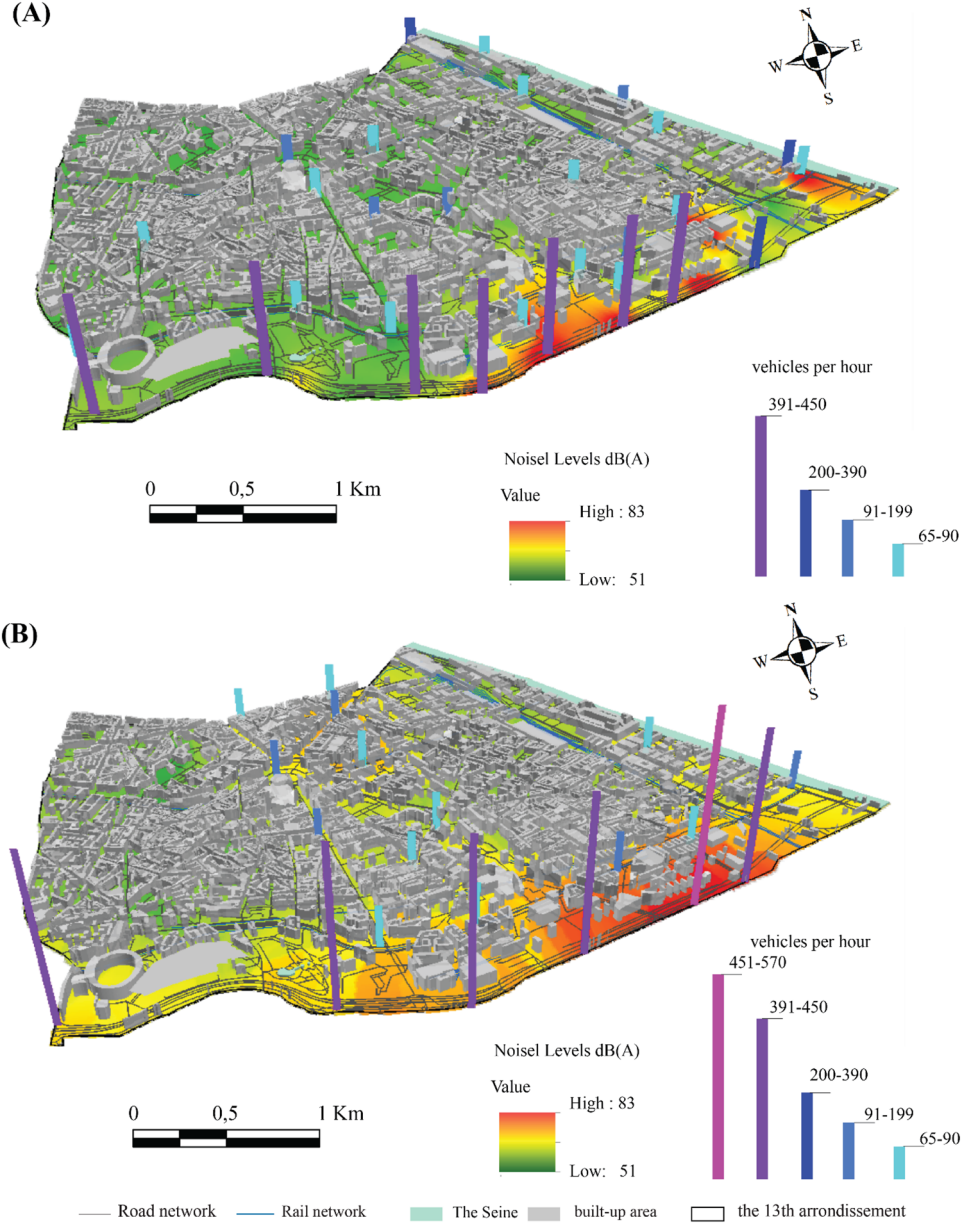
Spatial distribution of noise in the 13th arrondissement during the morning traffic peak, (**A**) during the lockdown, (**B**) outside the lockdown (average of 5 min per point, validated three times, measurements carried out between September 2020 and February 2022, time step 5 s, EXTECH sensor).

During the morning traffic peak during the lockdown periods, the noise level exceeds 76 dB (A) only at the Boulevard Périphérique due to the number of vehicles exceeding 400 cars/5 min, which is much higher than at the other sites (Fig. 11A). A few changes can be noted outside the lockdown: in addition to the Boulevard Périphérique, the noise level rises at the major intersections in the 13th arrondissement, such as Place d'Italie and Quai d'Austerlitz (Fig. 11B).

**Figure 12:**
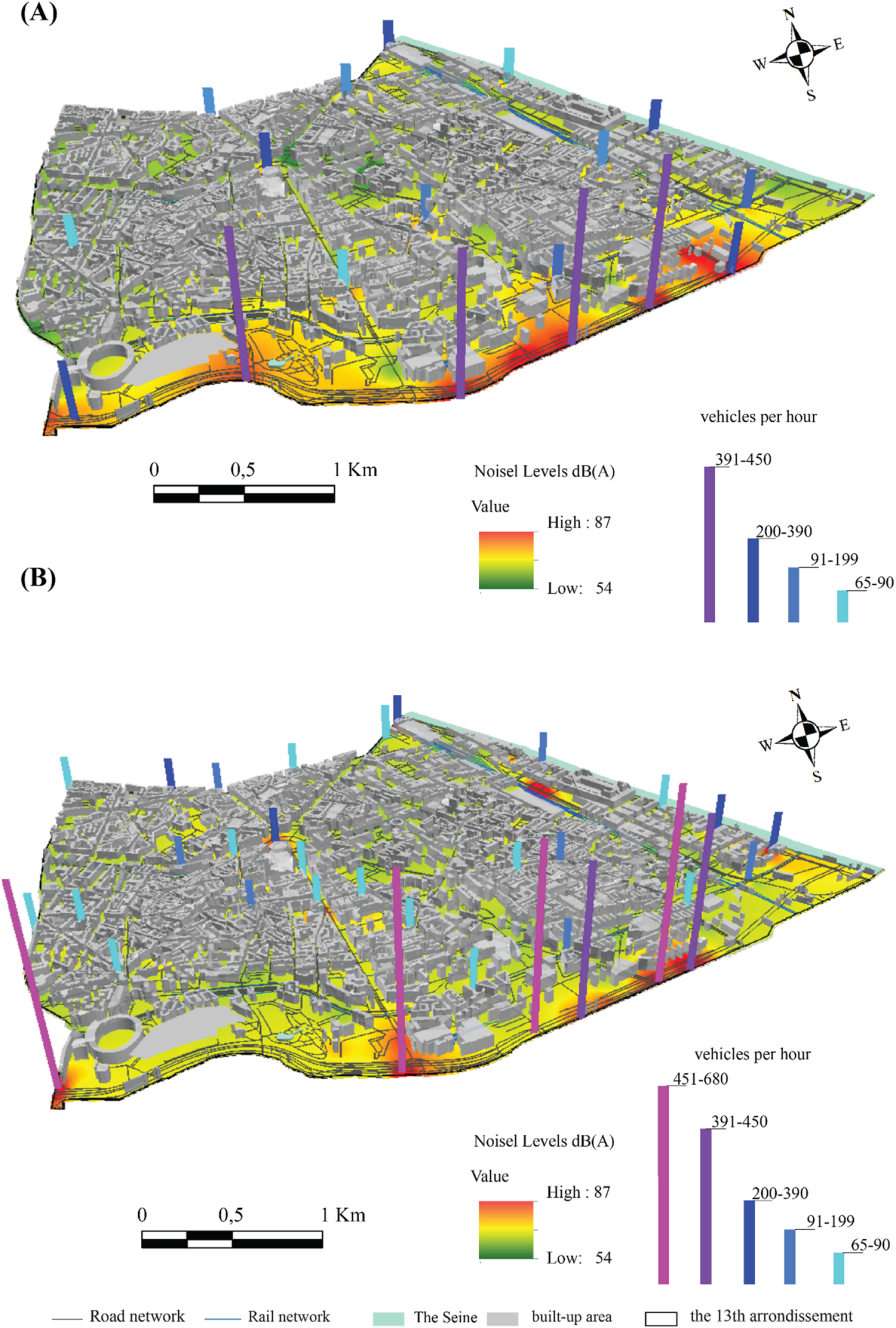
Spatial distribution of noise in the evening in the 13th arrondissement, (**A**) during the lockdown, (**B**) outside the lockdown (average of 5 min per measurement point, validated five times, measurements carried out between September 2020 and February 2022, time step 5 s, EXTECH sensor).

In the evening, during the lockdown periods, noise levels below 70 dB (A) were recorded near secondary roads, e.g. rue Bobillot or rue de la Glacière, while levels below 64 dB (A) were observed far from the main roads, e.g. rue Nationale (Fig. 12B).

Outside the lockdown, the density of traffic on the main roads facilitated the development of noise peaks located at the end of the major roads, for example, the intersections at Tolbiac, at the Place d'Italie roundabout, at the intersection linking the Quai d'Austerlitz to the Boulevard de l'Hôpital, where noise levels sometimes exceeded 82 dB (A) (Fig. 11B). Outside the junctions, along the main roads, values ranging from 72 dB (A) to 78 dB (A) were recorded, for example on Avenue d'Italie, Avenue des Gobelins and Quai Panhard et Levassor.

During the first weeks of the second lockdown ending, low values were recorded on the main roads, such as Boulevard August Blanqui and Boulevard Arago, with noise levels between 64 and 68 dB (A), due to the partial resumption of transport. Similarly, shopping centres remained closed and teleworking was widespread. The difference in noise levels between the Boulevard Périphérique and the other main and secondary roads can be explained both by the greater volume of traffic, the engine speeds, and the use of higher speeds on the Boulevard Périphérique. Commercial vehicles, which are more frequent on the Boulevard Périphérique and run-on high-pressure diesel, are particularly noisy, unlike biogas vehicles such as buses and city cars, which are more frequent outside the Boulevard Périphérique (Fig. 11B). This uneven distribution of noise levels in the 13th arrondissement of Paris can be explained by the variability of road traffic and the number of lorries. The 75th percentile of the number of lorries ranges, on average, from 5 lorries/5 min for the lockdown period to 9 lorries/5 min outside the lockdown.

In the evening, the noise level increases slightly compared to the morning. Some changes can be noted in the spatial configuration: certain areas that are busier in the evening stand out, such as the Avenue de France and the François Mitterrand library. There is a considerable difference near the cafés and cinemas compared to the lockdown period (Fig. 12). The study of the spatial and temporal variability of noise pollution mainly highlighted the particularity of the 2020 lockdowns. A slight decrease in noise pollution was observed during the lockdowns due to a considerable reduction in road traffic density. Relatively low noise levels were observed except for the Boulevard Périphérique recording an excess of 8 dB (A) during the lockdowns and 13 dB (A) outside the lockdowns compared to the average in the 13th arrondissement (Fig. 12B). These results are consistent with those of Bruitparif^24^ concerning the first lockdown (Fig. 12A).

Despite the importance of rail infrastructure in the study area, the influence of rail on noise levels remains low, as most railroads are underground.

## Discussion

Mobile measurements have made it possible to quantify noise levels in the various districts of the 13th arrondissement and to delimit areas where noise pollution is high. Vulnerability studies are now possible in areas exposed to this hazard. Compared with the Bruiparif study, based on data from 5 stations only, all located NW of the study area, our work provides more detail on the spatial scale. They provide original information, as the Bruiparif study did not present data for the 13th Arr in 2020. In future work, it will be possible to show the difference between the 2020 and 2021 lockdowns by analyzing the results of our mobile measurements.

The results obtained concerning the drop in noise levels during the covid 19 period in the 13th arrondissement of Paris are representative of what's happening on the scale of the whole agglomeration and other big-sized cities in the Europe. It can possibly be taken as an example also for other western industrial-ized countries.

As is the case with studies carried out in Europe, in large cities, noise has decreased during peak hours. The reduction in noise levels was greater in active areas than in traffic-dominated areas. For instance, the lack of people on the street in the Grands Moulins university campus (Université Paris Cité) explains a considerable drop in noise.

However, even during the lockdowns, high noise levels were recorded in the vicinity of construction sites such as the Paris Rive Gauche campus, Avenue de France, Boulevard Général d'Armée Jean Simon and Quai Panhard Levasseur. This part at the north-eastern end of the 13th arrondissement has been marked by many construction sites, such as the gigantic project of the two asymmetrical towers "Tours Duo" next to the Boulevard Périphique in the south-eastern corner of the 13th Arr. The objectives were largely achieved, since the IDW interpolations are consistent with the field observations which, although not exhaustive, enabled us to understand the spatio-temporal variation in noise pollution. The errors associated with the limits of this mapping method such as the correlation with noise explanatory factors are not detailed here but will be the subject of future work based on a simulation using multiple linear regression to predict noise by combining the following explanatory factors: the number of vehicles, the proximity of crossings, the proximity of construction sites, the number of road lanes, the morphology of arterial roads.

Because our measurements were taken during the evening from 5 to 7:30 p.m., our study does not show the noise peak observed at 8 p.m., which coincided with the clattering of pots and pans with which Parisians thanked and paid tribute to the healthcare staff and other essential workers. In Madrid, for example, the applause with which the people thanked the healthcare staff was accompanied by a peak in noise pollution^27^.

## Conclusion

In the 13th arrondissement, the COVID-19 lockdown caused a substantial decrease in mobility, affecting noise levels. This work was conducted to describe the effects of lockdown measures on noise levels and to analyse the spatio-temporal variability of noise levels based on 4200 points, during peak hours. The results of the study show a significant reduction in noise levels in the city during the lockdown. Decreases in noise levels are recorded during the pandemic closure period, but these improvements were only between 1 and 10 dB (A). On the Boulevard Périphérique, however, the decrease was very low, contrary to our expectations, probably due to the number of vehicles exceeding 400 cars/5 min. The changes identified in noise levels due to the pandemic are significant and can be used to inform the approach to noise management. For example, the implications of the development of remote working in the context of the health crisis also relate to lifestyles and therefore to the mobility habits of employees (commuting).

This study showed that, outside of lockdown periods, noise pollution can penetrate hundreds of metres into fully residential areas, e.g. the Butte aux Cailles district. A difference is observed between the morning and evening periods. The average reduction obtained is between 4 and 5 dB (A) during the morning on working days and exceeds 7 dB (A) in the early evening. Apart from the main roads and crossings, a fairly high noise level could be observed in the vicinity of the construction sites, which were allowed to operate despite the lockdown. A significant increase in the noise level in the evening during the second lockdown period is recorded towards the Ivry Olympiade sector, which has a high population density. This was due to people going out into the urban open space, as the lockdown rule in France allowed for a one-hour outing. Experiences from the pandemic offer insights into better practices and policies for urban transport development in the 13th arrondissement of Paris.

Other future work, such as noise modelling, will enable us to propose, if possible, solutions to reduce noise peaks. This can be done by introducing noise-reducing pavement^41,42^.

Future work should take into account the risk aspects of noise exposure, by cross-referencing areas with noise peaks with data on population vulnerability, such as age and standard of living.

## Data Availability

The datasets used and/or analysed during the current study available from the corresponding author on reasonable request.
